# Qualitative Evaluation of the Impact of a School Mental Health Literacy Curriculum on Student–Teacher Relationships

**DOI:** 10.3390/bs14080649

**Published:** 2024-07-27

**Authors:** Kristen Figas, Tucker Chandler, Madison Niles, Brooke Chehoski, Brittany Parham, Mark D. Weist

**Affiliations:** 1 Department of Psychology, University of South Carolina, 1512 Pendleton Street, Columbia, SC 29208, USA; etc3@mailbox.sc.edu (T.C.); chehoski@sc.edu (B.C.);; 2 Department of Psychiatry, University of Maryland School of Medicine, 655 W. Baltimore Street, Baltimore, MD 21201, USA

**Keywords:** mental health literacy, universal support, school mental health, student-teacher relationships

## Abstract

Mental health literacy (MHL) programs, which aim to improve knowledge, reduce stigma and promote help-seeking behavior, are a promising approach to meeting the growing mental and behavioral health needs of youth. This study aimed to understand the relational impacts of a MHL curriculum on students and teachers. A MHL curriculum was delivered in middle school classrooms across 11 schools in two diverse school districts in the Mid-Atlantic and Southeast regions. Fifteen teachers and counselors who delivered the MHL curriculum participated in focus groups to describe their experiences using the curriculum and perceptions of its impact. Qualitative focus group data were analyzed via team-based inductive thematic analysis following a grounded theory approach. Findings indicate that educators perceived the universal school MHL program to have a positive impact on relationships amongst students and between students and teachers. Participants reported that the MHL curriculum helped to open conversations about mental health and related topics by developing common language and providing an opportunity to model vulnerability. Having these conversations improved classroom rapport and helped teachers develop deeper connections with students. As a result, teachers and students achieved greater empathy and students advocated more for themselves and their peers. Implications for integrating MHL programs into multi-tiered frameworks in schools to expand access to mental health supports are discussed.

## 1. Introduction

Child and adolescent mental health has been declared a national crisis in the United States, with multiple high-ranking U.S. officials and federal agencies citing the need for urgent attention from multiple sectors [1,2,3,4]. Prior to the COVID-19 pandemic, more than one-third of children and adolescents were considered high risk for developing a diagnosable mental health disorder in the future [5]. The COVID-19 pandemic has compounded these concerns, leading to increased stress, anxiety and depression, substance use, and acting out behaviors in recent years [1,6,7,8]. Considering the scale of child mental health concerns and their impact on academic and life outcomes, schools have faced increasing pressure to address mental health [9]. School mental health services, wherein mental health clinicians are embedded in schools to provide on-site therapy to students, have long been promoted as a potential solution [10]. By reducing access barriers, school mental health services greatly expand the availability of mental health services for children and youth [11]. However, given long-standing mental health workforce shortages [12], focusing on individual therapy remains an unlikely solution to meeting the significant demand for mental health support. This is particularly true in the context of the COVID-19 pandemic, which simultaneously increased mental health needs and disrupted access to traditional mental health services [13].

Universal approaches, like school-based mental health promotion, are a promising alternative. By providing supportive programming to the entire population of students, as opposed to targeting only those already presenting significant mental health concerns, universal programs aim to improve mental health and prevent the development of future mental health challenges for all students. Universal mental health promotion takes a variety of forms and may focus on building social-emotional skills, increasing mental health awareness, or preventing behavior problems, to name a few [14]. Mental health literacy (MHL) programs are one such option. MHL is defined as “knowledge and beliefs about mental disorders which aid their recognition, management or prevention” [15]. This includes recognizing signs of distress and disorders, understanding causes and risk factors, knowledge of options and processes for seeking help for mental health challenges, and attitudes toward risk factors and help-seeking [15]. It follows, MHL programs aim to develop knowledge, attitudes, and skills to support mental health. Inaccurate knowledge of mental health and mental illness and stigma are known barriers to children and adolescents recognizing and addressing their mental health concerns [16,17]. MHL programs directly target these barriers by increasing knowledge of mental health and mental disorders, reducing stigma, and promoting help-seeking behavior [18,19]. Although conceptually similar to social emotional learning (SEL), SEL focuses on learning about and managing emotions and social interactions and does not include the same emphasis on mental health challenges and help-seeking that characterizes MHL [20].

School-based MHL programs have been demonstrated to improve mental health-related outcomes for students and teachers alike. MHL programs delivered to youth are associated with improvement in adolescents’ knowledge of mental health, attitudes toward mental illness, and help-seeking behavior [21,22,23,24,25]. In turn, this improvement in mental health literacy predicts adolescents’ psychological well-being [26]. Some research also suggests that mental health literacy programs improve adolescents’ sensitivity and empathy, increase prosocial behavior, and reduce conduct problems among teens [27]. Similarly, MHL programs delivered to educators are associated with improvements in teachers’ knowledge and attitudes, helping behavior and confidence addressing mental health concerns of their students, as well as reductions in teachers’ psychological distress [28].

Although these findings are encouraging, there is still relatively little extant research evaluating the effectiveness of school-based universal MHL programs, particularly within the United States. Existing research evaluating school MHL programs is also almost exclusively quantitative, largely constrained to self-reported knowledge, attitudes, and help-seeking behavior. There is a dearth of qualitative research exploring these or other benefits. Moreover, little is known about educators’ perceptions of these benefits and their experiences delivering MHL curricula. Perceived outcomes or advantages contribute to motivation, thus influencing the likelihood of implementing a program or intervention [29]. If educators do not perceive tangible benefits, they may be less likely to implement MHL programs, regardless of actual outcomes. Thus, understanding educators’ perceptions of the impacts of MHL programs is critical to successful implementation and sustainment of this form of support.

### Present Study

Given the limited research examining school-based MHL in the United States and the importance of understanding implementers’ perceptions, the purpose of the present study is to examine teacher perceptions of the impacts of an educator-delivered school-based MHL program on students and teachers. This inquiry was guided by the following questions:

1. What are educators’ perceptions of the impact of an educator-delivered school mental health literacy program on teachers? What benefits and/or challenges did educators experience while delivering the MHL program?
2. What are educators’ perceptions of the impact of an educator-delivered school mental health literacy program on students? In particular, how does participating in a MHL program impact students’ behavioral health and well-being?

## 2. Materials and Methods

### 2.1. Design

This exploratory qualitative study occurred in the context of a cluster randomized controlled trial (RCT) funded by the Patient-Centered Outcomes Research Institute (PCORI) comparing traditional school mental health services (i.e., mental health clinicians providing evidence-based therapeutic services within schools with limited integration or oversight) with enhancements targeting teaming, mental health literacy, and cultural responsiveness and equity. The sample of the parent study includes 22 middle schools in the Southeast and Mid-Atlantic United States. Half of the schools (*n* = 11) in each region were randomized to receive the enhanced intervention elements (herein referred to as the enhanced condition), including *the Guide* MHL curriculum [30]. The remaining 11 schools in the comparison condition implemented school mental health services without the enhancements listed above. The mental health literacy curriculum was not implemented in comparison schools. The five-year RCT was originally designed to include three years of implementation (intervention versus comparison) while student participants were in sixth through eighth grade and one year of follow-up after students matriculated to high school. However, due to school closures and prolonged disruptions associated with the COVID-19 pandemic, the implementation phase persisted for four years, with high school follow-up eliminated.

Following each school year, focus groups were conducted with clinicians and educators in each condition to collect qualitative feedback and strengthen implementation. The present study utilized data from focus groups conducted with teachers and counselors in the enhanced condition following the third year of implementation to evaluate progress during the previous years and inform adaptations to the MHL curriculum and implementation following this study’s completion. The protocol for this supplemental study was approved as exempt by the University of South Carolina’s Institutional Review Board.

### 2.2. The Guide

The Mental Health and High School Curriculum Guide (referred to as *the Guide*) is an evidence-based mental health literacy curriculum that has been widely implemented in high schools across Canada [30]. *The Guide* is targeted toward students aged 13–15 (grades 7–10) but is regarded as a flexible resource that can be adapted to different populations and settings. A downloadable version of *the Guide* is freely available in six languages, including two English dialects. The American English version of *the Guide* was implemented in the present study, though adapted by the research team for use with middle school students.

*The Guide* was originally developed for use in Canadian schools and evidence-based programs developed for specific populations often require adaptation when generalized to new cultures and contexts. The current study is the first randomized controlled trial in the United States to pilot this mental health literacy program. Thus, with permission from developers, *the Guide* underwent cultural adaptations to enhance content relevance, program acceptability, and ease of implementation within American middle schools. Cultural adaptation generally involves a systematic process of intervention modification based upon characteristics of the target population [31]. Program content, delivery, and procedural considerations are the most common cultural adaptations across frameworks [31,32]. Adaptations of *the Guide* prioritized content and program delivery changes and were refined using an iterative stakeholder feedback process.

Two study team members, a faculty and post-doctoral fellow, with school mental health expertise reviewed original content prior to year 1 of implementation and modified language level, concepts, and scenarios to ensure developmental appropriateness for 10–14 year-old students. Interactive learning tools (e.g., videos, handouts, PowerPoints) were also updated with media featuring similarly aged, racially/ethnically diverse peer examples and prominent cultural references (e.g., local sports teams). Pertaining to delivery adaptations, this study developed an optional teacher–clinician co-delivery model to increase teacher comfort, promote student engagement, support ease of implementation, and maintain intervention fidelity. Approximately half of the classrooms employed this approach in their facilitation of *the Guide*. At the conclusion of implementation year 1, participating clinicians, school counselors, and teachers provided intervention feedback during annual focus groups and their recommendations guided further refinement of the adapted MHL program.

*The Guide* targets four key components of mental health literacy: understanding how to promote mental health, understanding mental disorders and treatment options, reducing stigma, and improving help-seeking skills [30]. Five modules are delivered in order, beginning with building foundational knowledge of mental health before progressing to the brain’s role in mental health, stigma and mental illness, support and treatment, and strategies for maintaining positive mental health. *The Guide* was designed to be implemented by teachers in classrooms and includes detailed lesson plans, learning activities and materials, and knowledge checks to facilitate implementation. Live virtual trainings are available throughout the year at no cost to educators, and training resources, guidance documents, and Supplementary Materials are freely available online to further support implementation. Furthermore, *the Guide* creators also released a guidance document on adapting modules to virtual modalities during the COVID-19 pandemic school closures. For this project, most educators elected to discontinue use of *the Guide* during the transition to remote learning due to the inordinate challenges associated with an unexpected shift to online instruction. However, delivery continued once students transitioned back to in-person school.

### 2.3. Sample

Participants were recruited from a pool of 27 teachers and school counselors who delivered *the Guide* during the previous school year. The recruitment pool stemmed from the enhanced condition of the parent study, given that the primary focus was on the MHL curriculum implemented only in the enhanced condition. Purposive sampling was employed to identify individuals from whom the richest qualitative information about the study questions could be obtained [33]. In this case, the participants represented the individuals with the greatest experience both implementing the MHL curriculum and observing its impact on the school community. Teachers and counselors who did not implement *the Guide* were not included in the recruitment pool. A total of 15 individuals (56%), including 9 teachers and 6 school counselors, participated in the focus groups. Eight (53%) participants represented schools in the Southeast region, with the remaining seven (47%) representing the Mid-Atlantic region. The group was demographically diverse, with eleven (73%) female participants and nine (60%) Black or African American participants, with the remaining participants being male and White, respectively.

### 2.4. Procedure

Participants were invited to participate in one of two 90 min virtual focus groups using the Zoom platform [34]. Both focus groups were facilitated by the same study team member. All participants provided verbal consent to participate in the focus groups and were compensated with a USD 100 gift card. The same interview script was utilized during both focus groups. The following questions were posed to all participants:

1. How has delivering *the Guide* impacted your experience as an educator?
2. The past two years have brought some significant changes to the country (e.g., COVID-19, social unrest). How have you seen students’ behavioral health needs change or evolve?
3. How have your students been impacted by *the Guide* curriculum this year?
4. How have students’ relationships with one another or with their teachers changed?
5. How has implementing *the Guide* changed your relationships with students?
6. What have you heard students say about *the Guide* and/or mental health since they have been exposed to *the Guide*?
7. Do you view your role as part of the mental health system at your school any differently than you did before?

The focus groups were recorded and transcribed using the online platform’s automatic speech-to-text audio transcription feature. A research assistant reviewed the recordings and cleaned and de-identified each transcript in preparation for data analysis.

### 2.5. Analytic Plan

Qualitative data were analyzed via a team-based approach to inductive thematic analysis. Thematic analysis is a flexible and theoretically agnostic approach to qualitative analysis focused on identifying and categorizing codes to reveal themes and patterns between variables [35,36]. Qualitative coding can follow a deductive process, with data analyzed according to theoretically driven a priori codes, or an inductive process (e.g., grounded theory), in which case codes are developed and categorized over the course of the analysis process [37,38]. Due to the exploratory nature of the present study, inductive procedures were followed to generate novel codes that did not rely on pre-existing assumptions about expected findings.

The flexibility of thematic analysis has raised concerns about the accuracy, consistency, and trustworthiness of conclusions [35,36,39]. To mitigate these concerns, we used an iterative team-based approach aimed at achieving agreement across multiple coders. Having multiple coders independently analyze the data is recommended to enhance the credibility of qualitative findings [40]. Prior to coding, the authors developed a detailed coding protocol outlining the steps to the coding process and defining and exemplifying the types of codes to be tracked. The coding team was trained on this protocol and met regularly to share progress and debrief challenges. As recommended by Nowell and colleagues, detailed notes and records from the multiple rounds of coding were kept by the study team to create an audit trail documenting the full coding and theming process [39]. To further foster trustworthiness, team members met frequently during the coding process to engage in reflexivity regarding personal biases with respect to the research questions, review notes, and triangulate codes [39].

The coding protocol generally aligned with the six-phase thematic analysis process outlined by Braun and Clarke, with additional rounds of coding and review embedded within the process to accommodate the team-based approach [35]. First, coders developed familiarity with the data by reading through the transcripts and noting initial ideas. Next, they engaged in first cycle coding (i.e., open coding) employing a combination of elemental (e.g., structural, descriptive, and in vivo) and affective coding methods ideally suited to capturing participant experiences [38]. Structural coding assigns labels to segments of qualitative data encapsulating a concept or content area that is related to the research question [41]. For example, the code “relationships” might be assigned to a string of data speaking to a variety of ways in which relationships between individuals have changed in response to some event. In descriptive coding, coders assign a word or short phrase to identify the topic of an excerpt of qualitative data [42,43]. Examples include codes such as “students”, “teachers”, “knowledge”, or “advocacy” to convey topics discussed by participants. In vivo coding utilizes the language used by the actual participants to capture the meaning of a passage [44]. Finally, emotion codes label the emotional experiences conveyed by participants, such as “confusion” or “tension” [38]. Simultaneous coding, in which multiple types of codes can be applied to the same string of qualitative data, was permitted during the first cycle of coding; however, all conflicts and redundancies were resolved during the second cycle such that a given string of qualitative data could only yield one code [38].

A team of six total coders, all research assistants supervised by two senior coders, engaged in the open coding process. Each coder independently coded the entire data set (two focus group transcripts), and the team met regularly to review and compare codes and discuss challenges and discrepancies. After the full transcripts were coded independently by each coder, the team met to review all of the initial codes before progressing to the next round of coding. During this meeting, the coders identified conceptual similarities between codes, collapsing synonymous codes into single codes and discussing terminology to assign to codes. At this point, the two senior coders determined the final set of codes to be applied to the data.

During second cycle coding (i.e., axial coding), codes were categorized to “reassemble” the data [38]. During this phase, two senior coders independently re-coded the entire transcripts and grouped codes into themes before reviewing the themes to establish agreement. Inter-coder reliability was calculated via percent agreement and Cohen’s kappa during axial coding, and agreement was substantial (92%, κ = 0.79). A third coder provided feedback to resolve discrepancies until unanimous agreement was reached for every code and theme. During this phase, the final codebook was developed, which organized the qualitative data by code (without duplication) and defined all of the codes and themes.

## 3. Results

### 3.1. Opened Conversations

Teachers and counselors delivered *the Guide* to their classes with the goal of improving students’ MHL; however, the impact of this curriculum surpassed this singular goal. Almost unanimously, participants explained that delivering the curriculum created opportunities to have deeper, more meaningful conversations with students. This was one of the most common themes to emerge, with 18% of coded data aligning with this theme. One participant shared about the way *the Guide* helped to facilitate these conversations, stating, “They brought more of the social emotional learning into the class, and it helped with that communication piece. I had some [teachers] that some students normally wouldn’t connect with, but they ended up actually connecting with them, because they did encourage other conversations in the classroom other than content”. Similarly, another participant explained how delivering a MHL curriculum gave them the opportunity to foster relationships with students by talking about their mental health, stating, “There was one activity where it would talk about certain situations and if they would consider that mental distress or no distress or mental health problem, this and that. I think it’s kind of allowed for different conversations to be had with the kids. And some of the teachers would open up and share things about themselves”. Another participant summed up this shared sentiment of expanding conversations beyond classroom content, saying, “I think it’s allowed for more conversations with the kids… I always say that we do way more than just being a teacher”. Two major themes emerged as participants described the impact of these conversations: increased vulnerability among students and teachers and development of common language.

### 3.2. Vulnerability

Participants reported a notable increase in vulnerability in both student–student and student–teacher relationships while using the MHL curriculum in their classrooms, both during lessons and activities and after the lessons were complete. This was a common theme, reflected in 14% of the coded data. They explained that *the Guide* provided a natural foundation for these conversations by giving students a designated space to learn more about mental health. One participant shared, “I found that my students, because I was the one delivering it to them…felt like they had more opportunities to talk to me… in confidence… Because I was the one opening that door for them”. Similarly, another participant shared how they used the curriculum, explaining, “I would just think using it to foster relationships with kids helping them to really engage what mental health is”.

Among student–student relationships, participants reported that students were sharing more, were more open with their emotions, and were more willing to share their own mental health experiences and struggles. Two participants’ responses captured this increase in students’ vulnerability, saying, “I think that the kids were able to just maybe open up a little bit more about what they had experienced. And actually be able to put, like, kind of a name, or describe what they were feeling in sort of a better, healthier way” and “I think it gave my kids the safe place to share”.

Participants also saw an increase in openness and vulnerability within student–teacher relationships. One participant explained that delivering *the Guide* in their classroom helped them feel more at ease when having challenging conversations with students and that this increase in vulnerability was related to its delivery. Another concurred, stating, “Some of the teachers would open up and share things about themselves”, indicating that *the Guide* not only set the tone for teachers to feel more comfortable talking about mental health in general, but also provided the space to share their personal experiences and challenges with mental health.

### 3.3. Developed Language

Not only did *the Guide* create opportunities for deeper classroom conversations about mental health, it also provided students with specific language to more accurately describe their experiences. One participant explained, “I’ve noticed that teachers have stated that students have more language to kind of describe some of the things that they’re feeling, and is making it more open to where it’s not where it’s so taboo. I feel like us doing it as a whole school thing, it has changed the perspective for some of the students”. Another participant echoed this statement, expressing, “[*the Guide*] definitely helps kids with how to talk about certain things or how to talk about their feelings… a lot of times, they’ll just jump to saying pretty extreme things. I think it gave them tools to [use]”. In addition to students developing their language surrounding mental health, participants also noted an increase in teachers’ comfort with these conversations. One participant noted, “Our teachers and other staff members are more comfortable now, in knowing what to say and how to talk about [mental health], as opposed to being scared of, ‘if I have to mention this to students, what’s going to happen?’ [Students] are also more comfortable with it as well, which has been a huge positive impact”. As students’ knowledge of mental health and vocabulary surrounding the topic increased, one participant explained that some students expressed feeling more “validated and seen”. Thus, although developing language appeared less frequently than other themes (representing 7% of the coded text), participants highlighted the importance of developing language to talk about mental health as a foundational process undergirding some of the other relational changes described herein.

### 3.4. Improved Rapport/Deepened Connections

Several participants shared about the impact of the MHL curriculum on improving connections and rapport within school relationships, with 11% of the coded text representing this theme. Participants expressed that delivering the curriculum in their classrooms “helped to foster positive relationships with the students” and “helped me build bonds with my students and teach kids self-care”. One participant who reported a positive impact on rapport shared, “It has made the connections and relationships with students deeper and more meaningful. It has also made me think more about what I say, and how I do things in my room”. However, one participant mentioned a caveat, explaining that teachers who “bought into” *the Guide* seemed to develop stronger connections and rapport with their students compared to those who did not utilize it. Another participant reinforced the importance of building rapport with students by providing a contrasting example of s who did not feel as “bought into” *the Guide*. They explained, “I think they had a hard time connecting with them, as opposed to some of our younger teachers that are, you know, really built very good relationships with their kids. You could just walk in a room, and you just feel either the boredom or the tension, or you could tell when they’ve had a connection with their team of teachers”.

In addition to strengthening student–teacher relationships, participants noted the impact of *the Guide* on their relationships with school counselors and clinicians. This came up fairly frequently, appearing in 12% of the coded text. One participant expressed, “Since I have been administering *the Guide*, I’ve been working more closely with my school clinician. I didn’t do that at all before, so we got to really talk about what she does and what I do. And when situations came up, I had an easier outlet, right? I knew who to send the students to”. Another shared this sentiment, explaining, “It gave me more awareness of what the clinician does in the school setting, and also made me more aware of what a great impact and how many students that person reaches”. These deepened connections not only strengthened relationships between teachers and school counselors and clinicians, but also positively impacted students as their teachers gained an increased understanding of the mental health providers and referral process within their schools.

### 3.5. Increased Empathy/Understanding

Participants reported an increase in empathy, explaining that there had been significant growth in this area in both student–student and student–teacher relationships. This was the most common theme, representing 26% of the coded data. Participants shared various accounts of seeing increased empathy amongst students. Participants reported that students seemed to “gain empathy” and were becoming “more considerate and accepting” of their peers. One teacher expressed, “My students enjoyed the Guide, and they were glad they were exposed to it. They had a better understanding of what some of their friends may be dealing with”. Several participants specifically noted an increase in instances of students supporting one another, accepting and validating different opinions and points of view, and extending more compassion to students different from themselves.

Teachers also developed greater understanding of students’ mental health needs. As teachers’ understanding of mental health increased, their capacity for empathy grew. Participants shared that implementing *the Guide* changed their perspective on their students’ mental health struggles, saying, “[It] enlightened me to see that so many students could relate to the topics” and “It changed my perspective of the students and what they had and what they had been enduring”. As teachers delivered the curriculum to their classes, they became increasingly aware of the extent to which their students were struggling with mental health, signaling a need for more support in this area.

Similarly, participants noted a change in the way their students perceived them, explaining that delivering *the Guide* provided context for adults to share more about their lives outside of school. This seemed to expand their students’ understanding that teachers are people who can also experience challenges related to maintaining mental health. One participant summarized this increase in student empathy, saying of their fellow teachers, “They’ve mentioned that their students kind of see them as more of a person…I think this is giving them a tool to be able to see their teachers, as you know, humans with actual problems instead of a perfect teacher”. Additionally, one participant shared that delivering *the Guide* during the onset of the COVID-19 pandemic particularly opened students’ eyes to their teachers’ humanity due to the pandemic’s universal impact on mental health. Participants reported that this combination provided context for their students to see them as more than the singular dimension of teacher. One participant explained, “I just think, because the whole world was going through it, it made us look more like people to them… I feel like there was a little more empathy from students when staff had things come up because they’ve, we’ve been through similar things”. Another shared, “Students have been more understanding of all the teachers are going through”.

### 3.6. Increased Advocacy

Participants reported an increase in students advocating for themselves as well as their peers. They observed that students became more aware of their peers’ experiences with mental health, reached out to share their mental health experiences, and asked for support for themselves and others. One participant shared, “It allowed them to see what some of their friends may have been struggling with, and they gave them the insight, and it gave them the tools and resources of like, ‘Hey, I can’t help this person, like I may not completely understand it, but I can get you to someone who can.’” Another participant shared about how *the Guide* equipped students with the language to ask for help, saying, “I had a lot more students, I guess self advocating for themselves, like wanting to be referred to mental health more, or they you know what kind of said, ‘Do you think I have a problem?’ ‘I’ve been struggling with this, this, this, and this…do you think this can be anxiety or depression?’ You know, I’ve had them I guess questioning their own behavior, and reaching out for help if they, you know, truly needed it”. Others mentioned a general increase in hearing students talk about wanting to receive mental health services or worrying about their peers who they believed would benefit from such services. Ultimately, one participant shared, “[Students] have been more open about their emotions and willing to seek help”, highlighting the increase in both empathy and advocacy. Although expressed by several participants, increased advocacy was one of the less commonly occurring themes, reflected in 7% of the coded text.

### 3.7. Changed Role Perceptions

Finally, participants described a shift in students’ perception of their teaching roles in response to *the Guide*, explaining that students began recognizing teachers as a mental health resource. One participant explained, “We try to use *the Guide* and correlate it with finding a safe adult within the school, and to try to build more relationships amongst [teachers] and students, and just having students, to find who is that person that I could talk to and they know who their people are. Of course they know the counselors are there, they know the mental health counselors there. But there’s also other people within a school that you can go to that you feel safe and comfortable with”. Participants expressed that this shift was maintained during pandemic-related school closures, with one person expressing, “when [students] when through some struggles, they knew, even though they weren’t in the building with us, they could reach back out to me for support”. Overall, this was the least common theme, representing 5% of the coded text.

## 4. Discussion

While the primary goal of implementing a universal MHL curriculum was to increase knowledge and decrease stigma related to mental health, several themes emerged suggesting that educators perceived positive change in other domains. Educators reported observing an improvement in relationships amongst students and between students and teachers. They expressed that the MHL curriculum served as the foundation for facilitating deeper conversations about mental health and equipped them with both the tools and the confidence needed for initiating such discussions. These frequent, intentional conversations about mental health cultivated an environment of increased vulnerability, with educators modeling openness to their students by sharing their own mental health challenges. In turn, this increased vulnerability from educators provided space for both students and educators to discuss the universal impact of COVID-19 on mental health. The universal struggles brought on by the pandemic offered a new lens of empathy through which students began to view their teachers and seemed to change students’ perceptions of their teachers as a part of the mental health system at their school. With greater understanding of mental health and ways to seek support, educators observed growth in empathy and understanding across school relationships. Students appeared to demonstrate more supportive, empathetic behaviors towards their peers facing mental health challenges. Similarly, educators noted that the MHL curriculum both alerted them to the depth of their students’ mental health challenges and equipped them with the knowledge of how to connect students with resources outside of the classroom. Educators also noted increased rapport within their classrooms, attributing this factor to their level of “buy-in” to the program. Delivering the MHL curriculum simultaneously fostered an environment in which students gained knowledge and language to understand and express the struggles that they and their peers were facing, while learning how to advocate and seek help for themselves and their peers.

These findings extend existing knowledge by demonstrating that MHL programs may have important, indirect impacts. Previous research indicates that school MHL programs increase knowledge, decrease stigma, and improve help-seeking behavior, three variables directly addressed in *the Guide* [21,22,23,24,25]. As demonstrated herein, *the Guide* offers additional benefits beyond the objectives of the curriculum, like improving relationships and classroom dynamics. School climate and interpersonal dynamics, such as peer interactions and student–teacher relationships, are significant predictors of child and adolescent mental health [45,46,47]. The findings of this study suggest that universal MHL programs strengthen these critical protective factors while also building knowledge and skills, thus enhancing student mental health from multiple angles. These findings reinforce previous research suggesting that MHL programs increase sensitivity, empathy, and prosocial behaviors [27]. Naylor and colleagues demonstrated a quantitative increase in English adolescents’ sensitivity and empathy toward individuals with mental health difficulties, as well as a reduction in their use of pejorative language for mental health challenges, from pre- to post-intervention [27]. By extension, our findings suggest MHL programs may spark similar changes toward both peers and adults, regardless of their mental health status. Importantly, this qualitative analysis also offers insight into the process by which these and other changes occur. For example, the findings suggest that by providing an opportunity to discuss mental health and societal stressors, encouraging vulnerability, and developing common language, educators were able to connect with and understand their students on a deeper level, leading to shifts in educator-student dynamics.

These findings have added significance when contextualized with respect to the COVID-19 pandemic. The pandemic disrupted many school-level supports for students, precipitating decline in many students’ social, emotional, behavioral, and academic functioning [6,7,8,48]. By encouraging vulnerable conversations and providing an opportunity for students and educators to process societal stressors in relation to mental health, school MHL programs can facilitate recovery and improve classroom dynamics that were disrupted by the pandemic. MHL programs directly address mental health concerns exacerbated by the pandemic while simultaneously bolstering relevant protective factors (e.g., relationships), providing an efficient approach to addressing multiple needs schools currently face in the post-pandemic era.

MHL programs may be especially impactful when implemented as part of a multi-tiered system of support, such as the Interconnected Systems Framework [49]. Universal practices that promote student mental health, such as MHL programs, are an efficient, cost-effective approach for bolstering mental health supports and services. By addressing common barriers to service use and teaching coping strategies to promote well-being, MHL programs foster early response and prevent the escalation of existing concerns [50,51]. Moreover, MHL programs that are embedded into the school day and delivered to all students have the potential to reduce the prevalence and severity of mental health concerns, thereby enabling mental health providers to focus on meeting the most pressing individual needs. Thus, by augmenting preventive/promotive mental health support at Tier 1, MHL programs can strengthen the capacity of the school system to respond effectively at advanced tiers. MHL programs such as *the Guide*, which is freely available, packaged in a ready-to-use format, and designed to be implemented by traditional school personnel (e.g., teachers or staff), may be especially appealing to schools and districts seeking to expand the continuum of supports available to students without incurring prohibitive costs.

In addition to augmenting the continuum of supports, MHL programs provide an opportunity to strengthen critical systems processes like interdisciplinary collaboration. Bronstein’s interdisciplinary collaboration model describes five components foundational to effective teaming: Interdependence, Newly created professional activities, Flexibility, Collective ownership of goals, and Reflection on process [52]. Interdependence within interdisciplinary teaming involves reliance on collaborative professionals as it is more effective than isolated practice in achieving collective goals [52]. MHL programs delivered at the classroom level encourage teachers to regularly interact and collaborate with school mental health clinicians and other specialists (e.g., school counselors, social workers, and psychologists). Conversely, related school mental health providers implementing MHL in classroom settings must rely on educators to advise on educational structures, behavior management systems, and teaching methodologies to promote student engagement and learning. Newly created professional activities are co-developed products or structures building upon the unique expertise of collaborators [52]. Pertaining to MHL, newly created professional activities can take a variety of forms, such as development of diverse content facilitation models (e.g., in-person, virtual, hybrid) and coordinated adaptation of curriculum to match school context. Flexibility involves power sharing and role “blur” to meet the needs of the target population [52]. Traditionally, the educator role has been limited to facilitating student learning and growth specific to academics. Implementation of MHL warrants expansion of their role to supporting student development of mental health awareness, coping, and help-seeking resources. MHL collaboration also allows mental health professionals to engage in content coaching, curriculum training, teaching with students, and other mental health promotion and prevention roles. Collective ownership of goals illuminates the importance of shared responsibility in implementation and outcomes [52]. Shared responsibility may manifest as broad screening of student mental health needs, increased communication through referrals, or inviting greater discussion of mental health concerns in the classroom and during team problem solving meetings (e.g., MTSS, student support teams). Finally, Reflection on process refers to discussions of shared responsibilities and incorporation of feedback to improve partnership effectiveness [52]. Distribution and collection of pre–post assessments of mental health knowledge, help-seeking behaviors, stigma, and coping strategies can provide critical information for identifying programmatic strengths and opportunities for growth. Satisfaction surveys can also be used with program facilitators to assess their competence in delivering the intervention, as well as perceptions of content relevance, cultural responsiveness, and impact.

Taken together, implementation of MHL encourages interdisciplinary collaboration and may help to promote student well-being at the universal level, expand educators’ capacities for supporting student mental health, and increase the efficiency of behavioral health referral processes.

As demonstrated by interdisciplinary teams integrating the SEL curriculum in a rural school setting, it is likely that effective interdisciplinary collaboration also supports MHL adoption, implementation, scaling, and evaluation [53]. Regrettably, there is a dearth of research examining interdisciplinary collaboration with respect to school MHL program implementation to date. However, research does suggest that teachers recognize that they play a role in supporting students’ mental health, and also desire greater inter-professional collaboration to support their efforts [54]. Future research should explore bidirectional relationships between systems processes like teaming and collaboration and implementation of MHL programs, investigating interdisciplinary collaboration as both a determinant and outcome of school MHL program implementation.

## 5. Limitations

These findings should be considered alongside a few limitations. First, the participant sample from the present study is limited to teachers and counselors who volunteered to participate in the focus groups, restricting input from other stakeholders (e.g., other school staff, family members, and students). It is possible that other stakeholders would offer a discrepant perspective on the impact of mental health literacy programs. It is also important to note that the present study persisted throughout all stages of the COVID-19 pandemic and therefore cannot be viewed or evaluated separately from the pandemic’s global impact on mental health. Factors such as an increased general public awareness of the mental health crisis, student regression due to school disruptions and challenges at home, and other external variables may have impacted the discussions between educators and students related to mental health. As a result, the outcomes of the present study may not be strictly attributable to the MHL curriculum. Finally, a primary challenge to implementing any universal mental health program includes the varied levels of “buy-in” from students, teachers, clinicians, and school administration. Thus, any relational change associated with MHL curricula may be dependent upon factors that vary between schools, such as levels of readiness to change, internal support, and how the curriculum was organized and implemented.

## 6. Future Directions

School MHL curricula, such as *the Guide*, are an accessible, effective, and efficient resource for promoting youth mental health. That said, there is a limited body of literature examining the impact of school MHL programs in the United States, as well as research examining the impact of school MHL programs on outcomes beyond knowledge, skills, and help-seeking more broadly. As such, future research should aim to replicate these findings in other regions and with different student populations to evaluate the generalizability of these effects. Additionally, there is a dearth of guidelines or considerations for implementing school MHL programs. Future research should focus on understanding the elements of effective implementation and identifying structures and processes that promote implementation in a variety of settings. This might include investigating contextual barriers and facilitators, adaptations to content and delivery, implementation support mechanisms, the role of school leadership, and methods of delivery.

## 7. Conclusions

Mental health literacy curricula are an accessible and effective resource for augmenting schools’ Tier 1 supports to improve student well-being. School-based mental health literacy curricula, such as *the Guide*, promote mental health by improving students’ mental health knowledge, attitudes, and help-seeking behavior. The current study identified additional benefits, demonstrating that mental health literacy curricula also contribute to positive change in student–student and student–teacher relationships. Mental health literacy programs provide an opportunity to discuss mental health and stressors, develop common language, and encourage vulnerability, in turn fostering empathy and understanding, increasing advocacy, strengthening rapport, and deepening connections amongst students and between teacher and students, ultimately enhancing relationships in the classroom. By simultaneously building knowledge and skills and promoting critical protective factors, universal school-based mental health literacy programs are an efficient strategy for supporting all students’ well-being.

## Data Availability

The original contributions presented in this study are included in this article, further inquiries can be directed to the corresponding author.
